# Clinical Character of CASPR2 Autoimmune Encephalitis: A Multiple Center Retrospective Study

**DOI:** 10.3389/fimmu.2021.652864

**Published:** 2021-05-13

**Authors:** Xiaoxiao Qin, Huajun Yang, Fei Zhu, Qun Wang, Wei Shan

**Affiliations:** ^1^ Department of Neurology, Beijing Tiantan Hospital, Capital Medical University, Beijing, China; ^2^ China National Clinical Research Center for Neurological Diseases, Beijing, China; ^3^ Neurology Department, Beijing Friendship Hospital, Capital Medical University, Beijing, China; ^4^ Beijing Institute for Brain Disorders, Beijing, China; ^5^ Beijing Key Laboratory of Neuromodulation, Beijing, China

**Keywords:** Caspr2, autoimmune encephalitis, clinical character, retrospective study, treatment

## Abstract

**Objective:**

To examine the clinical characteristics of autoimmune encephalitis associated with the contactin-associated protein-2 (CASPR2) antibody.

**Materials and Methods:**

Medical records of all patients diagnosed with CASPR2 antibody-associated encephalitis were retrospectively analysed. Data regarding demographic features, neurological symptoms and signs, laboratory tests, imaging results, treatments, and prognosis were collected.

**Results:**

A total of 25 patients aged from 3 to 79 years old were enrolled in this study, with a median age of 43. Eight of 25 (32%) were female, and 17 of 25 (68%) were male. The median age of symptom onset was 42 years old with the course of disease from onset to hospital admission ranging from 2 days to 6 months (median was 17 days). Six patients (6/25) had fever as an onset symptom. During the course of disease, cognitive disturbance was the most common symptom, which was observed in 17 patients (17/25) in total. Eight patients (8/25) met the criteria for limbic encephalitis. Epileptic seizure occurred in six of these eight patients. Four patients (4/25) were diagnosed as Morvan syndrome. All patients were positive for anti-CASPR2 antibody in the serum (1:10–1:300). In six patients, antibodies were detected both in the blood and CSF (1:32–1:100). White blood cell (WBC) counts in the CSF were elevated in eight patients (8/25). The concentration of proteins in CSF increased in 10 patients (ranging from 480 to 1,337.6 mg/dl), decreased in seven patients (ranging from 23.2 to 130.5 mg/dl) and remained at a normal range in the other eight patients (ranging from 150 to 450 mg/dl). Abnormal electroencephalogram (EEG) activities included slow background activity and epileptic patterns. Abnormal signals in the bilateral hippocampus were detected by magnetic resonance imaging (MRI) in three patients presenting cognitive disturbance. In one patient who had limbic encephalitis, increased metabolism of bilateral basal ganglia and the mesial temporal lobe was revealed by PET-CT. Eleven of 15 patients receiving immunotherapy experienced varying degrees of improvement. Relapse occurred in four of 25 patients (4/25) after 2 months.

**Conclusion:**

CASPR-antibody-mediated autoimmune encephalitis is characterized by diverse clinical manifestations. The most prominent conclusion revealed by this retrospective analysis is the involvement of both central and peripheral nerve systems, as well as a lower relapse rate, a good response to immunotherapy, and favorable short-term prognosis after treatment was also demonstrated. Besides, additional work is necessary to evaluate the long-term prognosis.

## Introduction

Autoimmune encephalitis (AE) is mediated by autoimmune response in the central nervous system (CNS), of which the clinical features vary with different autoantibodies. Autoimmune encephalitis was first recognized as early as 1968 when Corsellis et al. came up with the concept “limbic encephalitis” (1). In 2005, Vitaliani et al. reported a series of cases as “autoimmune encephalitis” for the first time (2). In 2007, Dalmau et al. firstly identified the so-called “anti NMDAR encephalitis”, by confirming the expression of autoantibodies against NMDAR on the surface of hippocampal neurons in such patients. These specific antibodies were known as NR1/NR2 functional threshold antibodies (3). The approach to diagnosis of AE was defined in 2016 by Graus et al. (4) With the deepening understanding of autoimmune encephalitis, more and more autoantibodies associated with AE were discovered, which makes the subgroups of AE more complex. In the past decades, a progressive discovery of antibodies against intracellular antigens such as Hu, Yo, and Ri (5, 6), glutamic acid decarboxylase 65-kD isoform (GAD 65) (7), and collapsin response mediator protein 5 (CV2) (8); extracellular synaptic proteins, such as leucine-rich glioma-inactivated 1 (LGI1) (7, 9); and cell surface antigens such as anti-*α*-amino-3-hydroxy-5-methyl-4-isoxazolepropionic acid receptor (AMPAR) (10), anti-receptor antibody encephalitis (anti-NMDAR) (3), and *γ*-aminobutyric acid encephalitis (GABAR) (11) has been reported. Among them, the voltage-gated potassium channel (VGKC) is a well-known membrane protein complex that was commonly bound by antibodies in AE. As parts of VGKC, leucine-rich glioma-inactivated 1 (LGI1) and contactin-associated protein-2 (CASPR2) could be detected in the immunoprecipitation with patients’ autoantibodies (12, 13).

CASPR2 is a cellular adhesion molecule (CAM) and part of the neurexin family (13). It regulates the formation of distinct axonal domains around the nodes of Ranvier, serving as a membrane scaffold that clusters Kv1 channels at the juxtaparanodal region (14). CASPR2 is a transmembrane protein with its C-terminal portion interacting with protein 4.1B—an ankyrin protein that may link the juxtaparanodal and paranodal adhesion complexes to the axonal cytoskeleton. It is located in neurons in the limbic system, basal ganglia, and other motor areas and sensation pathways and is rich in the temporal lobe, especially in the GAD65 positive inhibitory neurons (15).

Anti-CASPR2 antibodies have been reported in various clinical conditions (4) (**Supplementary Figure 1**). On one hand, the great variation is considered to be partly associated with significant variation in epitopes (1, 5, 6). On the other hand, the wide expression of CASPR2 in both central and peripheral nervous system allows various forms of involvement (16). Besides, there is an overlap between peptides of LGI1 and CASPR2. Thus, positive immunobinding of the autoantibody to both LGI1 and CASPR2 might be observed. This could explain the expanded phenotypes in addition to classical features of CASPR2 autoimmune encephalitis as clinical presentations are consistent with the antigenic localization and function of LGI1 and CASPR2 (7). However, the HLA DRB1*11:01 implicated in CASPR2 is not associated with LGI1.

The pathogenesis in anti-CASPR2 antibody-associated disease is believed to be due to the blocking action of the interaction between CASPR2 and Contactin-2 (6, 8) (**Supplementary Figure 1**) thus disrupting the expression of Kv1 channels. In some cases, decreased expression of Kv1 channels was found in areas such as dorsal root ganglia (9), and in some others, an increased expression of Kv1 channels was induced, especially in the inhibitory interneurons in the hippocampus (6–8). This might cause hyperexcitability and network disturbance that may lead to epileptic seizures (7), which was supported by neuroimaging studies (10).

The contactin-associated protein-2 (CASPR2) is a transmembrane protein which is located adjacent to VGKC on the cell membrane. The CASPR2-antibodies could be detected by immunoprecipitation on account of CASPR2 which is a site where most VGKC-antibodies bind to (13, 17), and CASPR2 plays a role in both the peripheral and central nervous systems (18). Therefore, we see patients with anti-CASPR2 encephalitis presenting limbic encephalitis (fever, epilepsy, amnesia, sleep disorder, hallucination, psychosis, behavioral disorder), Morvan syndrome (sleep disorder, hallucination, psychosis, behavioral disorder, constipation, tachycardia, hyperhidrosis, paresthetica, weight loss), and peripheral nerve hyperexcitability (paresthetica, fasciculation, limb twitch), which is rarely seen in other encephalitis. Moreover, the study of gene coding for CASPR2 has identified its role in neurodevelopmental disorders such as autism, intellectual disability, and epilepsy (15, 19, 20).

In conclusion, the great variability of syndromes associated with anti-CASPR2 antibodies, despite its rarity, should be noted in clinical practice as a differential diagnosis of conditions, such as limbic encephalitis and epilepsy, especially in older patients, newly onset psychiatric symptoms; neuropathic pain; and idiopathic ataxia, as immunotherapy has been identified to be associated with improved outcomes in patients with CASPR2-associated encephalitis.

## Materials and Methods

### Standard Protocol Approvals and Patient Consent

This study was approved by the Ethics Committee of Beijing Tiantan Hospital, Capital Medical University. The study was conducted in accordance with the declaration of Helsinki, and all patients provided informed consent for the use of their medical records.

### Patients

Twenty-five patients were enrolled in our cohort as CASPR2 antibody-associated encephalitis, all with positive results for CASPR2 antibody in the serum. Meanwhile, the same positive results were reported in part of the CSF sample as well. Our patients were collected from multiple centers, including Beijing Tiantan Hospital, Henan Provincial People’s Hospital, The First Affiliated Hospital of Zhengzhou University, Nanjing Brain Hospital and the Affiliated Hospital of Ningxia Medical University. In this study, we retrospectively analyzed their clinical features, including present and past history (epilepsy, tumor, autoimmune disease), manifestations, laboratory tests (including antibody titer), electrophysiology and imaging examinations, treatments, and prognosis.

All the patients included in the study met the criteria as follows: (1) Subacute onset (rapid progression of less than 3 months) of one or more of the ten major groups of manifestations, including psychosis, memory deficit, speech disturbance, seizure, movement disorder, loss of consciousness, autonomic dysfunction, and central hypoventilation; or Morvan syndrome(2)with or without CSF pleocytosis, encephalitis MRI features or EEG epileptic or slow-wave activity; (3) cerebrospinal fluid (CSF) or blood serum antibody testing positive for AE antibodies based on a cell-based assay (CBA) (FA 112d-1 for NMDAR, AMPAR, GABAbR, LGI-1, CASPR2; FA 1151 for LgLON5; Euroimmun Ag, Lubeck, Germany); (4) reasonable exclusion of other disorders.

### Laboratory Tests

Serum and CSF tests for autoantibody were routine for each patient suspected of autoimmune encephalitis. The spectrum of specific antibodies included D-aspartate receptor (NMDAR), leucine-rich glioma-inactivated 1 (LGI1), GAD65, contactin-associated protein-2 (CASPR2), *α*-amino-3-hydroxy-5-methyl-4-isoxazolepropionic acid receptor (AMPAR), and *γ*-aminobutyric acid type B (GABAB). Serum or CSF antibody tested positive for AE antibodies based on a cell-based assay (CBA) (FA 112d-1 for NMDAR, AMPAR, GABAB, LGI-1, GAD64, CASPR2; Euroimmun Ag, Lubeck, Germany). CASPR2-positive patients were enrolled with dilution of antibodies (1:10, 20,1:32,1:100, *etc.*). Patients who were then diagnosed with viral encephalitis were excluded. We conducted a complete immunological evaluation, consisting of anti-neuronal antibodies [Hu, Yo, Ri, amphiphysin, CV2/CRMP5 and paraneoplastic antigen MA2(PNMA2)], autoantibody series, anti-neutrophil cytoplasmic antibodies (ANCA), anti-nuclear antibody spectrum, anti-cardiolipin antibody, rheumatoid factor, anti-streptolysin O; serum level of complement was also tested as complement is involved in immune response and inflammation which might underlie autoimmune encephalitis. Thyroid function analysis included total T3 (TT3), total T4 (TT4), free T3 (FT3), free T4 (FT4), thyroid-stimulating (TSH), thyroglobulin (TG), anti-thyroglobulin antibody (TG Ab), anti-thyroperoxidase antibody (TPO Ab), and thyrotropin receptor antibody (TRAb). All patients accepted tumor screening by detecting tumor markers, and anti-neuronal antibodies. Whole-body PET-CT was completed in only five patients due to the limitation of individual financial capacity, medical insurance policies, and examination ability of some centers.

### EEG and Imaging

A video electroencephalogram (VEEG) was undertaken using EEG-1200C (Nippon Optoelectronics Corporation), EEG-V32 (Natus, Nicolet Corporation), EMU40EX (Natus, Nicolet Corporation), and Micromed-SD (Brain Quick, Micromed). Magnetic resonance imaging (MRI), with or without gadolinium injection, was undertaken using an NT 3.0-T Philips Gyroscan (Eindhoven, the Netherlands), GE-SIGNA (GE Healthcare), Vantage Tian 3.0 T (Toshiba, Japan), and Magnetom Lumina (Siemens Healthineers). In addition, 18 F-FDG positron emission tomography (PET) images were acquired using a PET/CT scanner (Elite Discovery, GE HealthCare, Fairfield, Connecticut, USA).

### Outcome Evaluation

The modified Rankin scale (mRS) was adopted for outcome evaluation. For all patients, except a 3-year-old and an 8-year-old, the Montreal Cognitive Assessment (MOCA) and the Mini-Mental State Examination (MMSE) were used to measure cognitive impairment. MMSE<24 or MOCA<26 was defined as cognitive disturbance.

### Literature Review

An extensive literature search was performed for the terms “CASPR-2” and “encephalitis” from January 2007 to October 2020. The reported results were reviewed and summarized. The primary search identified 94 topic-related publications on PubMed, and any studies and case reports that included more than five patients were included. Ultimately, five studies and case series were reviewed in discussion (more details in **Supplemental Table**).

### Data Availability

Anonymized data not published within this article will be made available by request from the principal investigator, WS.

### Statistical Analysis

SPSS 19.0 was used for statistical analysis. Descriptive statistics were applied to analyze clinical data, such as medians and percentages.

## Results

### Demographic Features

A total of twenty-five CASPR2-AE cases from 22 centers in China were collected for this study. Twenty-five patients aged from 3 to 79 years old with a median age of 43 were recruited into our study. The median age of symptom onset was 42 years, with the course of disease from onset to hospital admission ranging from 2 days to 6 months (median was 17 days). Eight of 25 (32%) were female, and 17 of 25 (68%) were male.

### Clinical Manifestations

Data for symptoms presenting at onset and during the course are summarized in **Table 1**. Six patients (6/25) had fever as an onset symptom, with the temperature exceeding 39°C in two patients (2/25). Five patients (5/25) had cognitive disturbances and complained of visual hallucinations, memory deterioration, behavioral disorders or déjà vu. Four patients (4/25) had dyskinesia, and two patients (2/25) had seizures as presenting symptoms.

**Table 1:** Characteristics and clinical features.

Characteristics	Values
Sex, n (%)	
Male	17 (68%)
Female	8 (32%)
Age, y, median (range)	43 (3–79)
Course of disease, d, median (range)	17 (2–181)
Clinical syndrome, n (%)	
Morvan syndrome	4/25 (16)
Cerebellar syndrome	3/25 (12)
Presenting symptom, n (%)	
Cognitive disturbance	5/25 (20)
Fever	6/25 (24)
Myokymia and neuromyotonia	4/25 (16)
Seizure	2/25 (8)
Numbness	2/25 (8)
Sleep disorder	2/25 (8)
Others	4/25 (16)
Symptoms during course of disease, n(%)	
Cognitive disturbances, n (%)	17/25 (68)
Amnesia	5/25 (20)
Behavioural disorder	5/25 (20)
Hallucination	5/25 (20)
Psychosis	7/25 (28)
Epilepsy, n (%)	5/25 (20)
Peripheral nerve hyperexcitability, n (%)	4/25(16)
Sleep disorder, n (%)	6/25 (24)
Insomnia	4/6 (67)
Difficulty falling asleep	2/6 (33)
Autonomic dysfunction^a^, n (%)	6/25 (24)
Constipation	3/6 (50)
Tachycardia	1/6 (17)
Urinary retention or hesitation	4/6 (67)
Hyperhydrosis	3/6 (50)
Pain, n (%)	4/25(16)
Weight loss, n (%)	2/25 (8)

^a^TWO or more symptoms could present simultaneously in one patient. Double symptoms occurred in five patients.

During the course of the disease, cognitive disturbance was the most common symptom observed in 17 patients (17/25 in total). Among them, five patients (5/17) had amnesia, five (5/17) had behavioral disorders, five (5/17) had hallucinations, and seven (7/17) had psychosis. Two or more symptoms occurred in four patients. Five (5/25) patients had epilepsy. Two presented generalized tonic–clonic seizures, and one only had focal seizures with automatisms. Autonomic dysfunction occurred in six patients (6/25), three had constipation, four had urinary retention of hesitation, three had hyperhidrosis, and one presented with tachycardia. Moreover, weight loss was seen in two patients, and insomnia was seen in four.

Eight patients (8/25) met the criteria for limbic encephalitis, presenting with cognitive disturbance and mental or behavior disorders. Short-term memory impairment was most common (4/8), with MMSE scores of 25, 5, 26, and 26. Some experienced meaningless speech or mis-actions such as washing hands in the toilet or wearing the wrong clothes. Epileptic seizures occurred in six of the eight patients. Four patients (4/25) had Morvan syndrome, which is characterized by involuntary muscle cramping and stiffness accompanied by insomnia, hallucinations, and autonomic symptoms. Two of them experienced weight loss of 12 and 15 kg. Tumor screening showed slightly elevated tumor markers. Three patients (3/25) had cerebellum symptoms, and their chief complaints were walking instability. Relapse was recorded in four patients, among which three experienced both sleep disorders and epileptic seizures.

### Laboratory Tests

The results of laboratory tests and imaging are summarized in **Table 2**. Blood and CSF were obtained before treatment. All patients were positive for anti-CASPR2 antibody in CSF or blood. In 19 patients, the antibody was detected only in blood samples (1:10–1:300), while six patients presented a positive CASPR2 antibody in both blood and CSF (1:32). By analyzing white blood cell (WBC) counts and protein levels, we found evidence of inflammation. WBC counts in the CSF were elevated, ranging from 11 to 432 cells/ul in eight patients (8/25). Meanwhile, proteins in CSF decreased in seven of 25 (ranging from 23.2 to 130.5 mg/dl) and increased in 10 (ranging from 480 to 1,337.6 mg/dl). In eight patients, proteins in CSF were at normal level (ranging from 150 to 450 mg/dl). Seventeen patients received immunological evaluations, although not all of them finished all test items. Normal thyroid function was found in most patients, except one patient whose FT4 was elevated (17.1 pmol/L). Positive thyroid peroxidase antibodies (TPO Ab) and thyroglobulin antibodies (TG Ab) were detected in three of 15 patients. One patient was positive for RO-52 antibody, and another was positive for anti-streptolysin O. Nineteen patients underwent a tumor screening test, none of whom was previously diagnosed with a tumor. A slight elevation (within triple of increase) of tumor markers, including Carbohydrate antigen199 (CA-199), Human Growth Hormone (HGH), Carcinoembryonic antigen (CEA), Cytokeratin-19-fragment (CYFRA21-1), squamous cell carcinoma antigen (SCC), pro-gastrin-releasing peptide (ProGRP), and neuron specific enolase (NSE), was observed. Alpha fetoprotein (AFP), CA125, CA242, CA72-4, CA50, and Prostate specific antigen (t-PSA) were also screened but kept in normal range. Moreover, one patient was diagnosed with lung cancer by PET-CT scan without a positive tumor marker.

**Table 2:** Laboratory results and imaging.

Test profile	n (%)
CSF	
Normal	8 (32)
WBC counts **＞**10 cells/**μ**l	8 (32)
Protein **≥**450 mg/**μ**l	10 (40)
Protein **≤**150 mg/**μ**l	7 (28)
Immunological evaluation	
FT3 increasing	1/15 (6)
Positive TPOAb and TGAb	3/15 (20)
RO-52 antibody	1/15 (6)
ASO antibody	1/15 (6)
Tumor marker	
Normal	14/19 (74)
Abnormal	5/19 (26)
EEG	
Normal	6/17 (35)
Slow background	6/17 (35)
Epileptic discharge	5/17 (29)
MRI	
Normal	17 (74)
Bilateral mesial temporal lobe hyperintensity	3 (13)
Unilateral mesial temporal lobe hyperintensity	3 (13)

### EEG and Imaging

**Figure 1:**
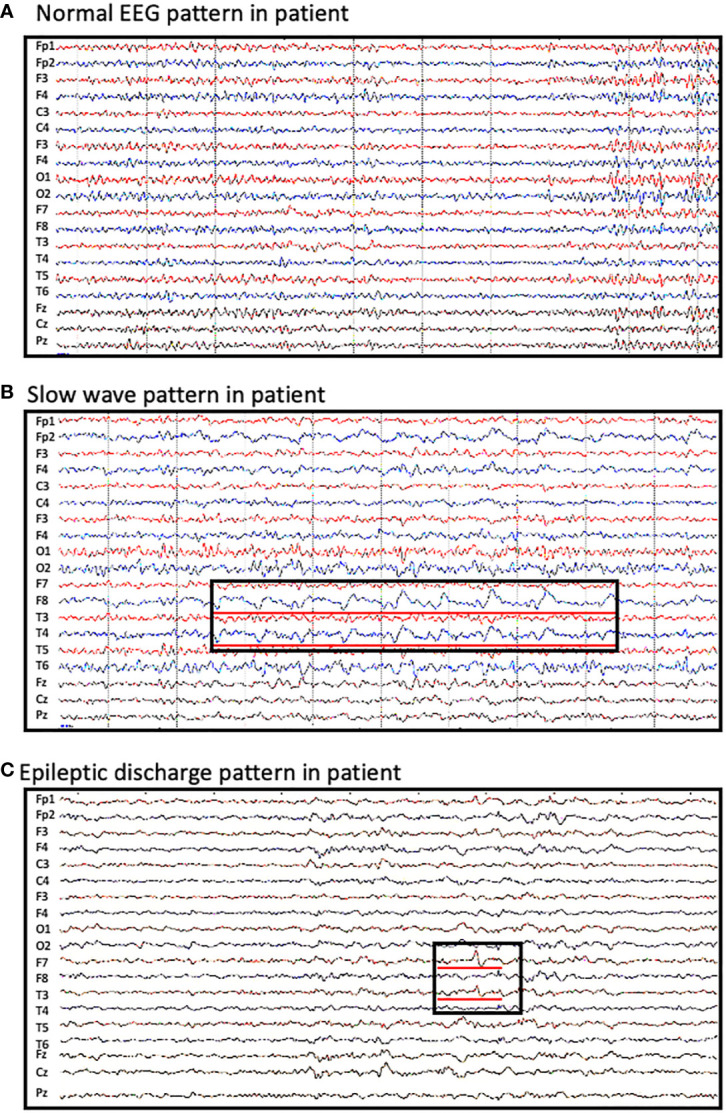
Abnormal EEG findings in patients with CASPR2 antibody-associated encephalitis. **(A)** normal EEG pattern observed in patients; **(B)** slow wave activities observed in F8 and T4; **(C)** interictal spikes and waves in F7 and T3.

Approximately 65% of patients reported an abnormal electroencephalogram (EEG) including slow background activity and epileptic patterns. **Figure 1** shows different EEG findings in patients with CASPR2 antibody-associated encephalitis. **Figure 1A** shows normal EEG patterns in patients; **Figure 1B** shows slow wave activities; **Figure 1C** shows interictal epileptic activities and epileptic patterns. One had interictal spikes and waves in the left hemisphere, as well as one event with spikes and waves with evolution in synchronous. Paroxysmal bilateral spikes and waves were recorded in another patient.

**Figure 2:**
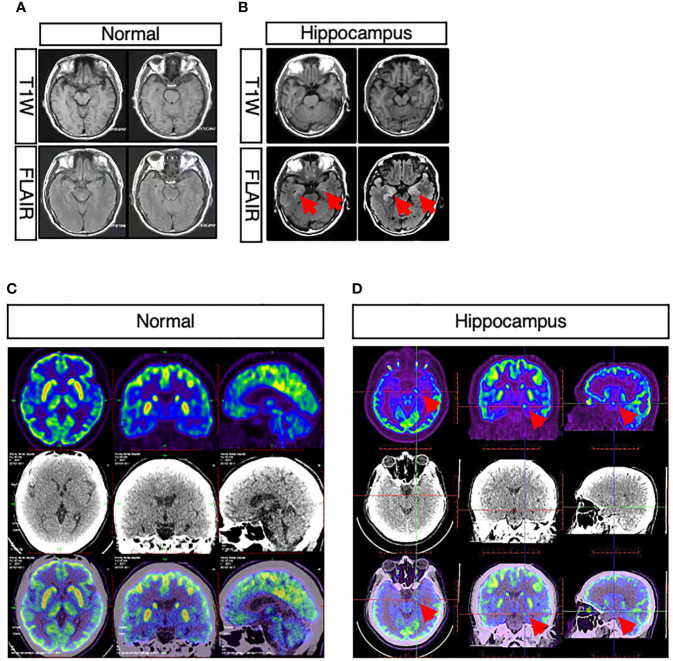
Abnormal neuroimaging findings in patients with CASPR2 antibody-associated encephalitis. **(A, B)** Comparison between normal and abnormal MRI showed abnormal signals in bilateral hippocampus in patients; **(C, D)** Comparison between normal and abnormal PET-CT revealed increased metabolism of bilateral basal ganglia and mesial temporal lobe in patients.

In three patients presenting cognitive disturbance, abnormal signals were observed in bilateral hippocampus (**Figures 2A, B** **)**, while in one patient, the right hippocampus appeared to be smaller than the left hippocampus. One patient presenting weakness in both lower limbs at onset had a remote infarction in the bilateral basal ganglia on MRI. Moreover, non-specific white matter disease and lacunar infarction were common, though not directly related to AE. In one patient who had limbic encephalitis, increased metabolism of bilateral basal ganglia and the mesial temporal lobe was revealed by PET-CT (**Figures 2C, D** **)**.

### Treatment and Prognosis

Immunoglobulin and corticosteroid were the most common therapy. Immunoglobulin plus intravenous methylprednisolone pulse (IVMP) was adopted in 15 of 25 patients. In our cohort, a standard protocol was intravenous immunoglobulin for 5 days, followed by steroid sequential therapy. Meanwhile, five patients (5/25) received IVMP therapy alone, while two (2/25) were given immunoglobulin alone. One of each of the other three patients was treated with circulation improving, anti-tuberculotic, and anti-viral therapies (**Table 3**).

**Table 3:** Treatment and outcomes.

Test profile	n (%)
Treatment	
Corticosteroids	5/22 (23)
IVIG	2/22 (9)
Corticosteroids + IVIG	15/22 (68)
Other treatment^a^	3/25 (15)
Outcomes^c^	
mRS ≤ 2^b^	6/17 (35)
At least −1 mRS point	11/17 (65)
No change on mRS	4/17 (24)
Relapse	4/25 (16)

a Other treatment including anti-viral, anti-tuberculotic treatments and vessel circulation improvement. These patients were not diagnosed as AE during hospitalization and thus given empiric therapy as above.

b The mRS scales of these patients were graded higher than 2 before treatment.

c Eight patients were lost to follow-up.

Comparing the modified Rankin scale (mRS) before and after treatment, 11 patients experienced varying degrees of improvement. For seven patients who were assessed as requiring external help in life (mRS ≥3), they were able to take care of themselves independently after treatment. However, six patients failed to respond to therapy, including one who remained severely disabled (mRS = 4). No patient reported disability progression. Three patients underwent lumber puncture again after treatment, showing decreased protein and WBC in CSF after treatment. Relapse occurred in four of 25 patients (4/25) after 2 months. Insomnia and epileptic seizures both appeared in three of them (**Table 3**).

## Discussion

We retrospectively analyzed 25 patients with confirmed anti-CASPR2 antibody-associated encephalitis in five centers. In our study, male patients accounted for a larger proportion, and they were older than females when initial symptoms appeared (median at 44 *vs* 38 years old). This result is similar to those of other studies in Asia (21). As known, autoimmune encephalitis is characterized by acute or subacute onset. Our patients had a median course of 17 days before admission, which might imply that CASPR2-associated encephalitis has a relatively short course of progression. However, we did notice that patients enrolled in our study went to the hospital sooner than those in other reports (21, 22).

The initial manifestation at onset varies by patient. For instance, one patient first suffered from right upper limb numbness, and another presented blurred vision as an initial symptom, which may be identified as cerebrovascular disease at the beginning. This could hinder timely and accurate diagnosis. An animal-model study demonstrated that CASPR2 was widely and deeply expressed in the cortex and involved motor and sensory pathways and the limbic circuit (23). This may explain the diverse symptoms observed.

During the course, muscle cramps or myokymia was one of most common symptoms and was recognized as peripheral nerve hyperexcitability (PNH). An *in vitro* experiment found that CASPR2 tends to express in inhibitory neurons (24), which means anti-CASPR2 antibodies can result in neuronal excitation through combination with its target. Among PHN syndromes, Morvan syndrome is notably special, not only because of its close specificity to Anti-CASPR2 antibody but also because both the central and peripheral nervous systems are involved (15). For instance, memory loss, sleeping disorder, and psychosis are typically seen in Morvan syndrome and no other PNH syndromes, such as Isaacs syndrome or cramp-fasciculation syndrome (25). Interestingly, in our study, the incidence of Morvan syndrome was 4/25, which is relatively lower than that in other studies (22). It may be attributed to the fact that our patients attended hospital and received medical intervention earlier, which suggests that initiating treatment early might be rewarding.

In our study, five patients (5/25) claimed to present epileptic seizures during the course of disease. This incidence is lower than that previously published, which was reported to be 30% by Husari and 53% by Agnes van Sonderen (26, 27). However, we found abnormal EEGs with epileptic seizure activities in five patients, while 11 patients received EEG examinations in this study. Considering that the information about seizure occurrence was based on the patient description and only a limited number of patients underwent EEG examination, there might be inevitable recall bias and missing report of non-motor or subtle seizures which were hardly noticed by themselves. Two types of seizures were reported in our study: generalized tonic–clonic seizure (2/5) and focal seizure with or without impaired awareness (3/5). No patients presented more than one type of seizure in the acute stage, which is in contrast to Morano’s report (28). Subclinical seizures occurred at a relatively higher rate, which motivated us to consider that widespread use of EEGs might be beneficial to the diagnosis of AE and epilepsy. The relapse rate is 4/25, lower than previously published data (27, 29, 30). More patients might be lost to follow-up owing to our calculation for relapse based on those who are re-hospitalized. Antiepileptic drugs help control seizures but allowed for relatively easy relapse in our study and others (31).

Additionally, tumors usually present as a comorbid to autoimmune encephalitis, mostly in anti-GABAB encephalitis (21). In our study, only one patient (1/25) had a lung tumor, which was confirmed by imaging. Although tumor markers were slightly elevated in some patients, it was not sufficient to suggest any malignancy. Meanwhile, tumors are also believed to trigger AE (32); however, in this study we did not find such an association.

Immunotherapy was initiated once the diagnosis was confirmed. Intravenous immunoglobulin (IVIG) and/or corticosteroid were applied to 22/25 patients. The response rate was relatively satisfying compared with previously published data (27, 33). Moreover, plasma exchange and rituximab were also proven to be efficient in some large cohorts (34), while a small cohort on LGI-1 encephalitis did not achieve an effective result (35). As a second-line drug, rituximab was not used in our cohort, possibly because of the good response to first-line therapy. Patients suffered from functional impairment in acute stage, while more than a half obtained favourable outcome after intervention (mRS ≤ 2) (**Table 3**).

To our knowledge, the incidence of autoantibody encephalitis is low and compared to that of NMDA, lgI1 or GABAB, and anti-CASPR2 antibodies are rarely detected. We first analyzed a retrospective cohort of anti-CASPR2 encephalitis in a Chinese population. Moreover, our study is based on a multi-center population. For comparison, we screened published cohorts about anti-CASPR2 encephalitis to date and then listed those with integrated clinical data in (**Supplementary Figure 1**). First, the spectrum of manifestation was not much different although previous studies did not describe much about the initial symptoms. For completeness, we included this in our study and noticed that the first presentation could be fairly varied. Second, second-line therapy was seldom applied to our patients, and by treating with corticosteroid and IVIG, they received a good prognosis as well.

## Conclusion

Autoantibody encephalitis is a relatively rare disease that lacks widely accepted international guidelines. We present this retrospective analysis of CASPR2 antibody-associated encephalitis to identify its clinical features. It is characterized by diversity of manifestation and involves the central and peripheral nerve systems and has a lower relapse rate. A good response to immunotherapy and favorable short-term prognosis after treatment was demonstrated in this study, but additional work is needed to evaluate long-term prognosis.

## Data Availability Statement

The original contributions presented in the study are included in the article/**Supplementary Material**. Further inquiries can be directed to the corresponding authors.

## Ethics Statement

The studies involving human participants were reviewed and approved by the Beijing Tiantan Hospital ethics committee. Written informed consent to participate in this study was provided by the participants’ legal guardian/next of kin.

## Author Contributions

XQ, HY, and WS wrote the initial draft of the manuscript, provided both figures, and made preliminary revisions. FZ, WS, and QW made crucial revisions to the manuscript. All authors planned the manuscript, critically revised the initial draft, and made final improvements prior to submission. All authors contributed to the article and approved the submitted version.

## Funding

This work was supported by the National Key R&D Program of China (#2017YFC13075), Capital Healthy Development Research Funding (2016-1-2011), Beijing Postdoctoral Research Foundation (ZZ 2019-09, 2020-06), China Postdoctoral Science Foundation (No.2019M660719), Beijing-Tianjin-Hebei Cooperative Basic Research Program (H2018206435), and Beijing Natural Science Foundation (Z200024).

## Conflict of Interest

The authors declare that the research was conducted in the absence of any commercial or financial relationships that could be construed as a potential conflict of interest.

## Supplementary Material

Supplementary Figure 1 Represents CASPR2 localization and its interaction with Contactin-2 forming the voltage-gated potassium channel complex (VGKC complex). The data show the variety of clinical phenotypes involving anti-CASPR2 antibodies and its overlap between central and peripheral nervous system syndromes.
