# Supplementary figures and images for: Clinical Character of CASPR2 Autoimmune Encephalitis: A Multiple Center Retrospective Study
Source: Front Immunol. 2021 May 13;12:652864. doi: 10.3389/fimmu.2021.652864 (PMC8159154; doi:10.3389/fimmu.2021.652864)

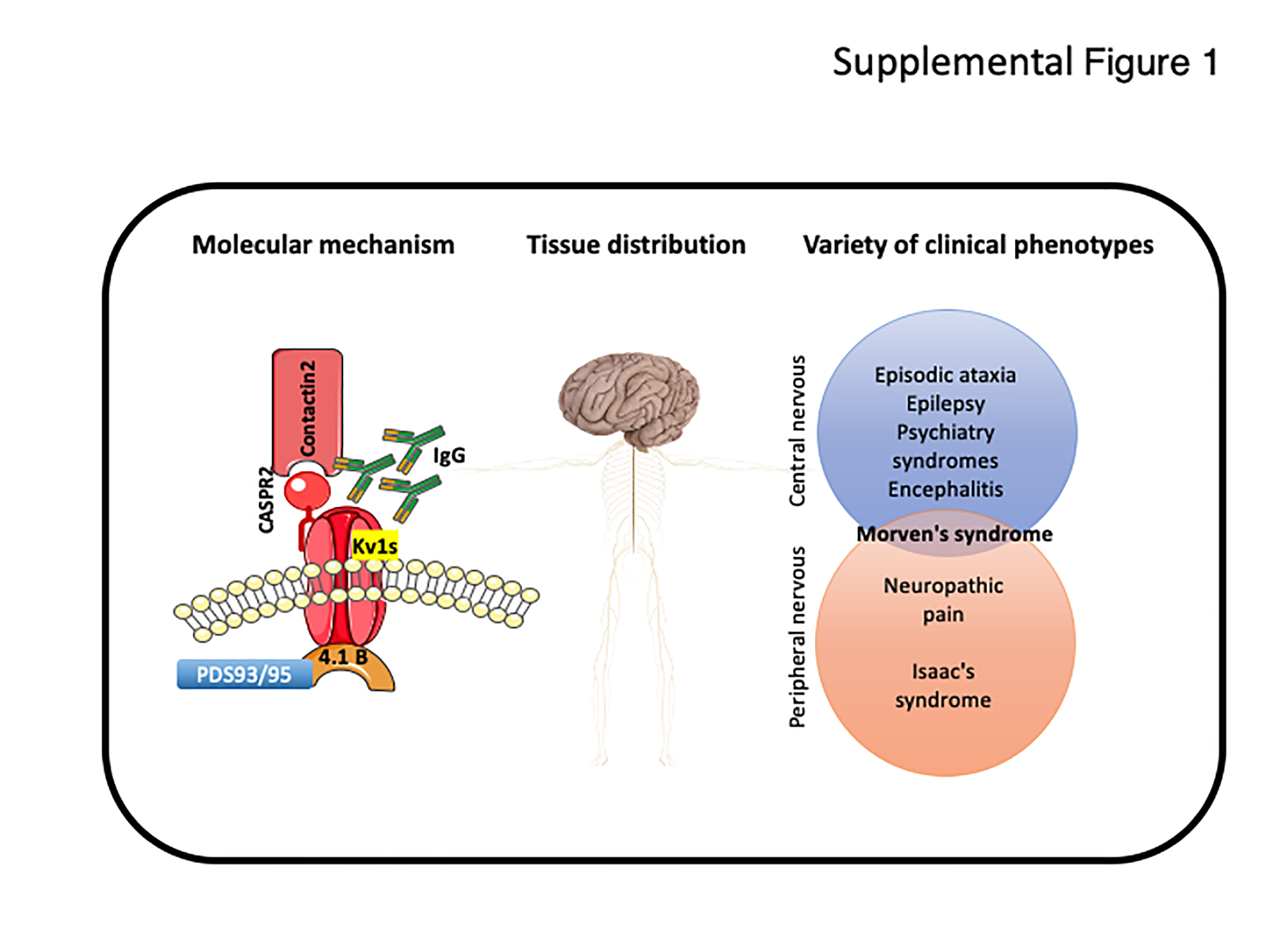

Supplement: Supplementary Figure 1 — Represents CASPR2 localization and its interaction with Contactin-2 forming the voltage-gated potassium channel complex (VGKC complex). The data show the variety of clinical phenotypes involving anti-CASPR2 antibodies and its overlap between central and peripheral nervous system syndromes. [file Image_1.tif]
