# Supplementary material for: Clinical Character of CASPR2 Autoimmune Encephalitis: A Multiple Center Retrospective Study
Source: Front Immunol. 2021 May 13;12:652864. doi: 10.3389/fimmu.2021.652864 (PMC8159154; doi:10.3389/fimmu.2021.652864)
Supplement: Supplementary file 2 [file Table_1.docx]

**Supplemental Table .** Comparison of several representative cohort or case series of CASPR2-associated autoimmune encephalitis

| #Of patients  (centres) | Demographic information | Clinical characteristics | Auxiliary examination | treatment | Prognosis and outcomes |
| --- | --- | --- | --- | --- | --- |
| C. G. Bien et al (multi-centre)^[1]^ | N=22 | Limbic encephalitis (18/22)  Morvan syndrome (2/22)  hyperkinetic movement disorder (2/22)  Seizures（18/22）  memory problems（13/22）  Affective disorder（5/22）  Neuromyotonia （2/22）  Movement disorder（3/22） | MRI: Persistence of FLAIR/T2 signal increase:(3/15)  Transgression into hippocampal atrophy: (2/15)  EEG：N/A | Steroids were most often used, but 3/4 of the patients required more than one immunotherapeutic. | mRS > 2 (5/19)  Immunotherapy response (–1 mRS point) ：（12/19）  Seizure-free for≥2 months：(8/13) |
| Eric Lancaster et al. (multi-centre)^[2]^ | N=8 | Movement disorder (5/8)  memory problems (3/8)  seizure (5/8)  psychosis (3/8)  autonomic dysfunction (2/8)  paraesthesia (2/8)  cerebellar signs (1/8) | MRI: Mesiotemporal hyperintensities:(2/7)  EEG: epileptic discharges:(1/5)  Sharp waves：（2/5） | First line: steroids (5/8)  IVIG：（2/8）  Second line:  Rituximab (3/8)  Cyclophosphamide (2/8)  Plasma exchange (2/8) | good prognosis:(5/8)  worsening symptom: (1/8) |
| Agnes van Sonderen et al. (2 centres)^[3]^ | N=38 | Limbic encephalitis (16/32)  Cognitive disturbances (30/38)  Epilepsy (19/36)  Peripheral nerve hyperexcitability (20/37)  Sleep disorder (19/38)  Autonomic dysfunction (14/32)  Weight loss (18/31)  Pain (20/33)  Cerebellar symptoms (12/34)  Tumour (7/37) | MRI: Hyperintensity medial temporal lobes (8/33)  EEG: epileptic discharges:(11/27)  Sharp waves：（9/27） | First line:  IV immunoglobulin:(13/30)  Steroids (18/30)  Second line:  plasma exchange (5/30)  Cyclophosphamide (2/30)  Rituximab (5/30) | favourable outcome (mRS≤2): (24/33)  died: (4/33)  relapse(≥1 year follow-up): (7/28) |
| Avi Gadoth et al. (single-centre)^[4]^ | N=51 | Seizure (21/43)  Limbic encephalitis (8/44)  Cognitive decline (17/44)  Personality change (5/15)  Sleep disturbance (5/15) | MRI: T2 mesial temporal hyperintensity (6/27)  EEG: epileptic wave (9/20) | corticosteroid was the most common initial treatment.  N/A in detail | N/A |
| Bastien Joubert et al. (single-centre)^[5]^ | N=18 | Cognitive disturbances (15/17)  Seizure (16/17)  Sleep disorder (4/17)  Frontal feature (10/17)  Cerebellar ataxia (4/17)  Mood disorder (3/17)  Sensory neuropathy (2/17) | MRI: hippocampal atrophy (3/15)  Temporomesail (12/15)  EEG: N/A | First line (IVIG, steroid): (17/17)  Second line  (CPA, MMF, rituximab, plasmapheresis) :(8/17)  AED: (2/17) | Relapse (6/17)  Favourable outcomes: (9/16) |

The mRS scales of these patients were graded higher than 2 before treatment

1. Bien C G, Mirzadjanova Z, Baumgartner C, et al. Anti-contactin-associated protein-2 encephalitis: relevance of antibody titres, presentation and outcome[J]. Eur J Neurol, 2017, 24(1): 175-186 [<http://dx.doi.org/10.1111/ene.13180>]

2. Lancaster E, Huijbers M G, Bar V, et al. Investigations of caspr2, an autoantigen of encephalitis and neuromyotonia[J]. Ann Neurol, 2011, 69(2): 303-11 [<http://dx.doi.org/10.1002/ana.22297>]

3. van Sonderen A, Arino H, Petit-Pedrol M, et al. The clinical spectrum of Caspr2 antibody-associated disease[J]. Neurology, 2016, 87(5): 521-8 [<http://dx.doi.org/10.1212/WNL.0000000000002917>]

4. Gadoth A, Pittock S J, Dubey D, et al. Expanded phenotypes and outcomes among 256 LGI1/CASPR2-IgG-positive patients[J]. Ann Neurol, 2017, 82(1): 79-92 [<http://dx.doi.org/10.1002/ana.24979>]

5. Joubert B, Saint-Martin M, Noraz N, et al. Characterization of a Subtype of Autoimmune Encephalitis With Anti-Contactin-Associated Protein-like 2 Antibodies in the Cerebrospinal Fluid, Prominent Limbic Symptoms, and Seizures[J]. JAMA Neurol, 2016, 73(9): 1115-24 [<http://dx.doi.org/10.1001/jamaneurol.2016.1585>]
